# What is new in Freezing of Gait?

**DOI:** 10.1007/s00415-026-13765-6

**Published:** 2026-03-21

**Authors:** Eileen Gülke, Simon J. G. Lewis

**Affiliations:** 1 Parkinson’s Disease Research Clinic, Macquarie Medical School, Faculty of Medicine, Health and Human Sciences, Macquarie University, Sydney, NSW Australia; 2 Department of Neurology, University Medicine Greifswald, Greifswald, Germany; 3 Department of Neurology, University Medical Center Hamburg-Eppendorf, Hamburg, Germany

**Keywords:** Freezing of Gait, Outcome measures, STN-DBS, PPN, Locus coeruleus, Adaptive DBS

## Abstract

Freezing of Gait (FOG) research is entering a rapidly evolving phase. Published in early 2026, the International Consortium for FOG (ICFOG) released updated clinical and technical definitions of FOG and introduced a consensus-based standardized testing protocol, the Giladi protocol (GP-FOG), which is currently still under evaluation in an ongoing study. Complementing these advances, two new outcome measures, the clinician-reported outcome (ClinRO) and the patient-reported outcome (PRO), are highlighted in this review. Recent technological developments in both detection and potential prevention of FOG are moving the field toward individualized, biomarker-based, on-demand treatment strategies. In addition, emerging insights into the pathophysiological mechanisms of FOG, particularly nondopaminergic contributions, are discussed. Together, these developments provide a contemporary framework for improving the assessment, understanding, and personalized management of FOG in Parkinson’s disease.

## Introduction

A substantial body of new work has emerged in the field of Freezing of Gait (FOG) in recent years, making an updated synthesis both timely and necessary. FOG is a common and highly disabling gait disturbance in Parkinson’s disease (PD), typically triggered during gait initiation, turning, or navigating narrow spaces, with around 80% of patients experiencing FOG across all disease stages [31]. In particular, FOG is strongly associated with reduced quality of life, an increased risk of falls and fractures, and greater loss of independence [59]. FOG is characterized by its paroxysmal and sudden onset, often described as a sensation of the feet being “glued” to the floor. Its episodic nature and clinical heterogeneity complicate experimental investigation, objective measurement, and reliable scoring in both clinical practice and research. These challenges have contributed to considerable heterogeneity in how FOG is defined, assessed, and reported across studies [25].

To address these limitations, the International Consortium for FOG (ICFOG) was established to bring together clinical and technical expertise and to develop a unified framework for FOG research. This review builds on these efforts by introducing the updated clinical and technical definitions of FOG, outlining a new testing protocol for accurately assessing FOG ‘on the fly’ and through video-based analysis, and presenting two upcoming rating scales: the clinician-reported (ClinRO) and patient-reported outcome (PRO). In addition, recent advances in technology-assisted assessment and treatment approaches are highlighted, reflecting the rapidly evolving landscape of FOG research and management. We conclude by reviewing current pathophysiological mechanisms, with a focus on nondopaminergic contributions.

## The clinical and technical definition of FOG

Why are two distinct definitions of FOG required, and what are the differences? The previously widely used definition proposed by Nutt et al. from 2011, describing FOG as *a brief, episodic absence or marked reduction of forward progression of the feet despite the intention to walk* [67], led to ambiguity in FOG classification, particularly with respect to episode duration and the presence of effective forward progression. The updated 2026 ICFOG *clinical definition* describes FOG as *paroxysmal episodes wherein there is an inability to step effectively, despite attempting to do so *[25]. A major advantage of this revised clinical definition is that FOG may occur during *any* gait-related stepping movement, including turning, step initiation, and walking in *any* direction, and can be easily applied in clinical settings. The *attempt to step* can be verified through self-report, observable body movements, or sensor-based measures, while the *inability to step effectively* is judged by the assessor relative to the individual’s own normal stepping pattern.

**Fig. 1:**
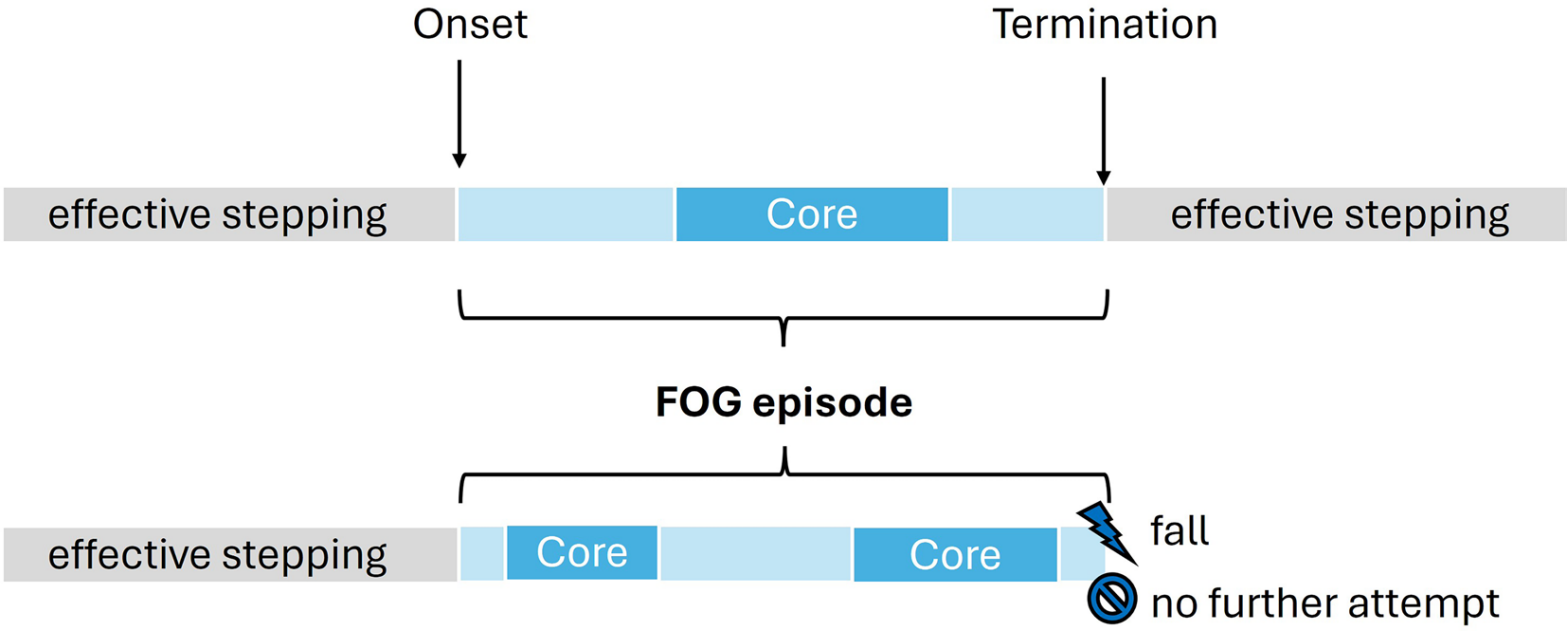
Visualization of the *technical* definition of FOG. A FOG episode begins with the first ineffective step or attempt to take a step and ends with 1) no further attempt to take a step, 2) two consecutive effective steps that resemble typical stepping performance, or 3) a FOG-induced fall. During the FOG episode, *core events*, referring to a complete cessation of movement, may occur. Based on Gilat et al. [25]

With regards to the *technical definition* of FOG, video scoring remains the gold-standard method for assessing FOG in clinical trials and research studies, typically quantified as the percentage of time spent with FOG in relation to the total task duration (%TF) [24]. Within the ICFOG framework, the onset of a FOG episode is defined as either the moment when any part of the foot lifts off the ground as part of the first ineffective step or the first attempt to initiate a step that does not result in any observable foot movement. A FOG episode ends with 1) no further attempt to take a step, 2) two consecutive effective steps that resemble typical stepping performance, or 3) a FOG-induced fall. Importantly, as FOG does not always involve a complete cessation of forward progression, the *core* of the episode, formerly referred to as *motor blocks* [22], may be additionally labeled and may occur once or repeatedly at any point during the FOG episode. The *technical definition*, therefore, distinguishes between FOG onset, FOG termination, the FOG *core*, and different FOG manifestations (Fig. 1). These manifestations include *akinetic, kinetic-trembling*, and *kinetic-no-trembling* FOG. *Akinetic* FOG is characterized by a complete cessation of lower-limb movement, whereas *kinetic-trembling* FOG is defined by rapid oscillatory movements. *Kinetic-no-trembling* FOG includes any other ineffective movements, such as paroxysmal shuffling or festination-freezing. Notably, continuous gait impairments, such as persistent shuffling or reduced step length, are not considered FOG under this definition. For further details and supplementary video material, please refer to Gilat et al., 2026 [25].

## The Giladi protocol to objectively measure FOG in clinic and research settings

In addition to an updated FOG definition, a standardized measurement protocol is essential, since current patient-reported outcomes are not sufficiently reliable, and existing FOG testing protocols diverge across studies [25]. A variety of clinical observation measures or instrumented gait assessments have used a range of different tools to assess FOG [56, 73, 94]. However, the utility of these approaches is constrained by several limitations, including insufficient standardization and gold-standard validation, extensive test batteries, no representation of common triggers of freezing (e.g., turning, dual-tasking, navigating doorways), as well as the impression that scores (e.g. the Ziegler FOG score) do not truly reflect clinical severity [30, 53]. To resolve these issues, a newly developed consensus-based FOG-provoking protocol, the *Giladi* protocol (GP-FOG), using a clinician-reported outcome (ClinRO) to quantify FOG severity in real-time, is currently being validated in an international, multicenter study (FOG-COA, NCT06519279). The *Giladi* protocol is named in memoriam of our dear colleague Nir Giladi, who made substantial contributions to the ICFOG and the development of these new definitions, the protocol, and outcomes scores. The test itself comprises a series of eight walking trials of varying complexity, as well as common FOG triggers, such as turning on the spot [5, 26] or walking through a doorway [2, 57]. Notably, walking in fast, short steps (“shuffling”) as well as full, rapid turns have been identified as the most sensitive ways to provoke FOG [66]. The GP-FOG consists of eight walking trials, including walking, dual-task walking, carrying a ball, turning, dual-task turning, shuffling around a box, box agility, which includes performing small forward, backward, and lateral steps around a ground-marked square, and walking through a doorway [55]. Dual-tasking involves performing the movement task simultaneously with a serial-3 subtraction task, i.e., counting backward from a randomly selected three-digit number in threes. The test is user-friendly, can be completed within 20 minutes, and does not require any additional equipment. The participants are asked to accomplish each task as quickly and safely as possible, while avoiding any strategies or techniques to prevent FOG.

## New rating scales: ClinRO & PRO

Along with the new *Giladi* FOG-provoking protocol (GP-FOG), a new rating scale, the *clinician-reported outcome* (ClinRO) is currently being developed by the ICFOG and is being validated in the FOG-COA study above. Current FOG rating scales fall short in rating the complexity of FOG: the MDS-UPDRS part III item 3.11. [29] is often used to evaluate the presence of FOG. Therefore, the participant walks for at least 10 m, turns 180°, and returns to the examiner, which is not a very sensitive approach to assess FOG [45, 66]. Although widely used, the Ziegler FOG score, covering 360° turns, doorway walking, and a dual-task (counting backward) with or without carrying water, also fails to differentiate between episodes of varying frequency or duration [30, 94]. Thus, there is a pressing need for a reliable clinical assessment outcome that can be widely implemented in both clinical and research settings to evaluate FOG severity, incorporating both frequency and duration of freezing episodes.

The *patient-reported outcome* (PRO) is also currently being developed and validated to evaluate the impact of FOG on a patient’s quality of life and to capture ‘the voice of the patient’, which will be critical when seeking FDA approvals for future therapies. This validation is also part of the ongoing FOG-COA study, described above. Current patient-reported outcome questionnaires, including the Giladi Freezing of Gait Questionnaire [23], the new Freezing of Gait Questionnaire (NFOGQ) [65], and the MDS-UPDRS part II item 2.13. [29] have limited validity and insufficient sensitivity to detect small effect sizes, rendering them unsuitable for clinical trials [37, 74]. The novel PRO that is being evaluated will potentially capture information based on the last 4 weeks from patients and their partners, addressing aspects of emotion, confidence, and self-worth, which could then be correlated with objective measures of FOG.

## Advances in technology to measure FOG

While ClinRO and gold-standard video-based ratings remain task-specific, emerging technologies offer the potential for more sensitive and comprehensive FOG assessments across both supervised and unsupervised settings. Notably, objective accelerometry-based measures provide a reliable quantification of freezing severity and mitigate the variability inherent with subjective clinical ratings [62].

Inertial measurement units (IMUs) are among the most commonly used and easily deployable tools for quantifying movement [55]. However, considerable variability remains in sensor number and placement, with current recommendations favoring five sensors in supervised laboratory settings and a single lumbar sensor for unsupervised assessments [55]. Despite their potential, IMU-based FOG detection is limited by the absence of a robust, validated analytical framework [55]. Moreover, unsupervised, continuous at-home IMU monitoring alone is not sufficiently sensitive to capture all aspects of FOG, highlighting the need to combine patient-reported outcomes, in-home FOG-provoking tests, and continuous monitoring to comprehensively assess FOG severity [15]. The Gait Advisors Leading Outcomes for Parkinson’s (GALOP) committee, an advisory committee for the Michael J. Fox Foundation for Parkinson’s Research (MJFF), recommended a minimum set of metadata to accompany standardized gait protocols, including pace, rhythm, asymmetry, variability, and turning measures [54].

The ultimate goal is to sensitively detect FOG in home-based environments, as PD patients’ gait performance in supervised laboratory settings often overestimates patients’ real-life gait abilities [36]. Besides, IMUs need to be permanently worn on the body, and the recording duration is limited by battery life [49]. One promising technology for continuous gait assessment is the potential use of a radar system where electromagnetic waves are transmitted, and the shift in reflected waves is measured, using the Doppler effect to measure the velocity of a participant. Radar provides multiple benefits, being independent of lighting, ensuring participant anonymization, and offering a cost-effective solution [87]. It was initially demonstrated to reliably capture gait characteristics, such as gait speed and step time, in a laboratory setting [87]. A three-node radar network featuring one torso node and two feet nodes has been shown to be the most adequate to extract spatiotemporal gait parameters in PD patients [49]. A recent study applied a low-power radar system in the homes of 50 participants, with and without PD, using a signal strength 1000 times lower than typical home Wi-Fi [46]. Combined with advanced signal processing and machine learning algorithms, this approach successfully assessed in-home gait. Strikingly, radar-measured gait speed correlated with PD severity, including MDS-UPDRS part III total scores, axial symptoms, and Hoehn and Yahr stage. Furthermore, the radar system captured disease progression by monitoring gait decline in a small sub-cohort of two participants over one year and motor fluctuations in response to levodopa intake, with gait speed increasing following medication administration [46]. These novel findings suggest the potential for automatic, wearable-free detection of freezing episodes in home environments.

## Advances in technology to prevent FOG

In FOG research, one important aim is to detect freezing episodes before they occur and ultimately prevent them. With emerging approaches, this objective is becoming increasingly attainable. In recent years, electrophysiological signals have been analyzed to identify a typical “freezing signature” with abnormal activity preceding and accompanying FOG episodes. These signals include cortical measures such as electroencephalography (EEG) and functional near-infrared spectroscopy (fNIRS), local field potentials (LFP), and kinematic signals, which may enable the development of multimodal systems for individualized monitoring and prevention of FOG [40].

Electrical activity of cortical neurons can be measured using EEG, which provides high temporal resolution, whereas fNIRS captures hemodynamic responses with better spatial resolution. During a Timed-Up-and-Go (TUG) task, EEG power in the alpha and beta bands increased in the central sensorimotor and occipital regions *before* the onset of freezing episodes, compared with normal walking, and the actual freezing episodes were associated with significantly increased theta and alpha band power within the central and occipital areas [10]. Furthermore, freezing episodes could be distinguished from voluntary stopping periods, which were associated with increased high-beta power in frontal, central, parietal, and occipital regions, as well as decreases in delta to low-beta power relative to FOG episodes. These findings were corroborated by fNIRS, which demonstrated that turning and doorway negotiation elicit cortical activity patterns resembling those observed during voluntary stopping, characterized by activation of the supplementary motor area (SMA) and prefrontal regions. In contrast, during freezing episodes, prefrontal cortical activation was reduced compared with voluntary stopping. Based on these observations, the authors hypothesized that freezing-provoking tasks may engage a stopping network with increased susceptibility to freezing [14]. However, due to the low signal-to-noise ratio of fNIRS and EEG, a large number of gait cycles must be recorded and analyzed using advanced algorithms to reliably distinguish gait-related from movement-related neural activity [55].

One of the major advances in Deep Brain Stimulation (DBS) technology in recent years has been the introduction of local field potential (LFP) sensing using the SenSight™ electrodes (Medtronic). LFPs reflect the summed postsynaptic activity of neuronal populations in the vicinity of the stimulation target. Within this signal, beta-band oscillations in the subthalamic nucleus (STN) have been consistently associated with motor impairment, particularly bradykinesia, and are modulated by dopaminergic and DBS therapy, with increased beta power serving as a marker of greater motor symptom severity [17, 42, 43]. Current DBS therapy uses continuous, 24/7 stimulation, which may lead to stimulation-related side effects such as dysarthria, stimulation-induced dyskinesia, or neuropsychiatric adverse effects [16, 80, 92]. By leveraging patient-specific beta-band activity, the newly available approach of adaptive DBS (aDBS) enables automatic adjustment of stimulation parameters based on predefined thresholds. However, movement-related LFP fluctuations [63] as well as ECG-artifacts [76] still remain challenging, complicating subsequent analyses. The successful implementation of aDBS still requires substantial clinical experience and research.

Despite overall improvements in global motor outcomes, gait worsening after bilateral subthalamic nucleus (STN) stimulation has been reported in approximately 42% of patients [85]. Furthermore, while DBS generally provides sustained long-term improvement in bradykinesia and tremor, gait performance often declines within the first five years post-implantation [77]. DBS programming troubleshooting strategies include low-frequency stimulation, lateralized subthalamic stimulation, and STN-substantia nigra costimulation [60] [47] [70, 90]. However, these approaches yield mixed results and are often associated with worsening of overall motor scores. A recent approach is to program aDBS for STN-DBS patients experiencing FOG [3]. In fact, a small pilot study confirmed the safety, tolerability, and feasibility of beta-burst driven aDBS for FOG [91]. Theoretically, identifying the FOG spectral biomarker in real time may allow DBS to be dynamically adjusted to either prevent a freezing episode or alleviate freezing episodes [55].

An alternative but well-established clinical strategy to prevent and overcome FOG is through the phenomenon of cueing [72]. Visual and auditory cues help focus attention on walking and shift gait control from habitual to goal-directed motor patterns [28]. In general, visual cues tend to improve stride length, whereas auditory cues primarily enhance cadence [78]. Externally generated cues can even improve gait performance during dual-task conditions [48]. Various, often expensive, cueing devices have been shown to improve gait, such as laser pointers [93] and laser-light shoes with foot pressure and inertial sensors [11]. Nevertheless, repeated exposure can result in habituation, reducing their long-term utility [28]. Moreover, no single cue is universally optimal, as effectiveness depends on individual factors such as cognitive function, and it remains unclear which patients benefit most from specific types of cues [27, 28]. Accordingly, the multicenter UNITE-PD study is currently investigating patient characteristics, changes in cueing efficacy, and the neural correlates of cueing [1]. Additionally, externally generated cues may be less effective than internally generated cues, such as singing [32], highlighting the need for more advanced cueing technologies [68] that integrate electrophysiological and kinematic signals. In line with the concept of aDBS, providing each patient with individually tailored, on-demand stimulation, adaptive cueing could offer similar benefits by responding to a patient’s unique ‘freezing signature’ and potentially preventing FOG before it occurs. Early approaches have shown promising results. For example, a novel on-demand cueing system using instrumented insoles detects FOG episodes via increased variability in step time, and delivers visual cues on demand, resulting in reduced freezing and increased walking speed [68]. Another innovative device, Cue2Walk, employs a single sensor worn on the lateral side of the shank below the knee to provide auditory and/or vibratory cues. Cueing can be triggered manually via a button press or hands-free gesture, such as a heel tap, or automatically, based on real-time detection of FOG episodes using a tri-axial integrated accelerometer and a specialized algorithm [84]. Notably, a model combining IMU and plantar pressure features accurately identified the shift from preFOG to FOG, highlighting its potential application in real-time FOG detection and adaptive cueing [69]. Additionally, analyses using fNIRS and EEG indicated higher parietal alpha activity during cueing in patients at later Hoehn and Yahr stages, which could be used as an electrophysiological signal to identify cueing responders [86]. Together, these findings further underscore the need for an individualized, biomarker-based cueing approach.

## Updates on FOG pathophysiology

The pathophysiology of FOG is still not well understood, but likely relates to a loss of automated motor control when there is a processing overload across disseminated neural networks, resulting in gait disturbances [83]. The *Neural Reserve* or *Cross-Talk Hypothesis* proposed by Lewis and Barker considers FOG as the momentary breakdown of normal gait, when there is an inability to concurrently process motor, cognitive, and limbic information, which overloads the system [44, 64]. The proposed model further asserts that in overcoming a freezing episode, the patient is able to suspend their performance of additional cognitive and limbic processes. By focusing on a goal-directed behavior, there is a reduction in the degree of over-activation within the output nuclei of the basal ganglia, allowing this circuitry to be ‘‘reset’’, once again facilitating movement. Thus, FOG appears to reflect a lack of neural reserve across shared pathways dealing with incoming information and functional output. A recent review from Tosserams and colleagues highlights the core pathophysiological principles of FOG: (1) loss of gait automation due to basal ganglia neurodegeneration with high inhibitory basal ganglia output, (2) degeneration of compensatory cortical areas, and 3) impaired integration of cerebral compensatory networks [83].

While dopaminergic therapies can improve FOG in some individuals, they do not fully ameliorate FOG symptoms [50]. Levodopa-unresponsive FOG suggests the involvement of nondopaminergic pathways, as does the clinical heterogeneity observed in patients with FOG [19]. Current evidence suggests that noradrenergic, cholinergic, and serotonergic systems might be involved in the pathophysiology of FOG. Beyond dopaminergic contribution, the role of the neurotransmitter noradrenaline has been recently implicated in FOG. An increase in arousal is associated [18, 52] and biologically linked to states of brain network integration implicated in this phenomenon [81, 82]. Effective integration of distinct and segregated brain networks critically relies on the noradrenergic ascending arousal system, which appears to be impaired to varying degrees in patients with FOG [58, 75, 88]. Widespread noradrenergic denervation has been observed in patients with FOG, particularly involving the Locus coeruleus and the thalamic noradrenergic system, both of which are closely linked to limbic circuits implicated in FOG pathophysiology [58]. Atomoxetine is a promising noradrenergic drug, as it is safe and well-tolerated in PD [71, 89]. It is a selective noradrenaline transporter inhibitor (SNRI), which prevents the cellular reuptake of noradrenaline, and is widely used for the treatment of attention-deficit hyperactivity disorder (ADHD) [41]. Atomoxetine has been shown to improve attention, response inhibition, executive functions, and anxiety in PD [89], which are all clinical features that have been associated with FOG severity. However, to date, only inconsistent reports of improvements in FOG have been reported in small samples [39, 71]. The Antifreeze trial (NCT07316296), sponsored by the MJFF, is a multicenter, single-dose, double-blind, placebo-controlled clinical trial with a cross-over design. The goal of this clinical trial is to investigate whether atomoxetine can reduce FOG and identify potential biomarkers for freezing responders.

Regarding cholinergic involvement, the striatum, the pedunculopontine nucleus (PPN), and the nucleus basalis of Meynert (NBM) represent the principal cholinergic sources in the brain and are affected by α-synuclein-related neurodegeneration [6]. Levodopa-unresponsive PD patients experiencing FOG have been shown to exhibit bilateral reductions in cholinergic terminals, predominantly in extrastriatal regions, using vesicular acetylcholine transporter positron emission tomography (VAChT-PET)[12]. Cholinergic deficits have also been demonstrated in patients with PD and a history of falls, suggesting a shared underlying mechanism for falls and FOG within a framework of impaired cognitive integration [7]. Therefore, the PPN has emerged as a promising DBS target, with small pilot studies reporting reductions in FOG and falls in subsets of patients [21, 61]. A currently ongoing clinical trial (NCT04605263) is comparing bilateral STN-PPN DBS with conventional STN-DBS. However, interim results from an exploratory randomized, double-blind, crossover trial did not demonstrate gait improvement after two months of combined STN-PPN DBS in patients with levodopa-resistant gait and balance impairments [9]. Pharmacologically, a randomized, double-blind, placebo-controlled phase II trial (ReSPonD) reported promising results with improved step-time variability in patients treated with rivastigmine, an acetylcholinesterase inhibitor [33]. Nevertheless, the results of the CHIEF-PD trial (NCT04226248), a multicenter, randomized, double-blind, placebo-controlled study evaluating rivastigmine for fall prevention, are still pending.

Apart from noradrenergic and cholinergic deficits, depression, a symptom commonly associated with FOG [20], is linked to the serotonergic dysfunction in PD [4]. Notably, reduced cerebrospinal fluid serotonin levels have been observed in advanced PD patients with gait disorders [38]. Clinical data on serotonergic drug effects on gait function remain limited. However, previously, ritanserin, a highly selective 5-HT2 receptor antagonist, significantly improved gait in 7 out of 10 patients in one small clinical trial [34]. Similarly, a short-term randomized, placebo-controlled 4-week trial found an improved walking speed in patients treated with paroxetine, a serotonin reuptake inhibitor (SSRI), compared to placebo [13]. With regard to FOG, functional MRI studies have revealed reduced functional connectivity between the dorsal raphe nucleus (DRN) and cortical structures, such as the supplementary motor area (SMA), a key region implicated in gait control and FOG [51]. Furthermore, preliminary evidence suggests that both selective serotonin reuptake inhibitors (SSRIs) and serotonin-norepinephrine reuptake inhibitors (SNRIs) may improve FOG [79].

In summary, nondopaminergic mechanisms underlying FOG may help delineate specific freezer subtypes and inform tailored noradrenergic, cholinergic, or serotonergic treatment strategies.

## Conclusion

Altogether, rapid progress in the field is enabling greater standardization in both clinical and research settings. The emergence of on-demand treatment strategies offers new hope for patients with Parkinson’s disease affected by FOG. Beyond identifying freezing episodes and developing potential interventions, the mechanisms underlying why and when nonfreezers transition to freezers remain poorly understood. Several risk factors have been identified and can be categorized into modifiable factors, such as more severe motor and cognitive impairment, and nonmodifiable factors, including older age and longer disease duration [35]. A recent machine-learning-based study further highlighted specific gait characteristics, including step length and stride width across both medication states, that are associated with freezing severity [8]. Understanding these predictors represents a pressing step toward elucidating the mechanisms behind freezing conversion and ultimately guiding targeted, individualized interventions.

## Data Availability

Data availability is not applicable to this article as no new data were created or analyzed in this study.

## References

[1] Albers C, Mirelman A, Avanzino L, Bloem BR, Botta A, van der Cruijsen J, de Lange E, Maidan I, Nieuwboer A, Pelosin E, Tosserams A, Weerdesteyn V, Gilat M, Nonnekes J (2026) Understanding cueing strategies for gait impairments in Parkinson’s disease: protocol of the multicenter UNITE-PD study. Eur J Neurosci 63:e70382 41562323 10.1111/ejn.70382 PMC12821084

[2] Almeida QJ, Lebold CA (2010) Freezing of gait in Parkinson’s disease: a perceptual cause for a motor impairment? J Neurol Neurosurg Psychiatry 81:513–518 19758982 10.1136/jnnp.2008.160580

[3] Anis S, Clark PA, Shaffer S, Hartwig E, Moser L, Scott J, Hennigs E, Walter E, Lopez Gonzalez T, Escobar D, Walter BL (2026) Introducing a workflow algorithm for adaptive DBS programming in Parkinson's disease. Parkinsonism Relat Disord:108199 10.1016/j.parkreldis.2026.108199 41577516

[4] Ballanger B, Klinger H, Eche J, Lerond J, Vallet AE, Le Bars D, Tremblay L, Sgambato-Faure V, Broussolle E, Thobois S (2012) Role of serotonergic 1A receptor dysfunction in depression associated with Parkinson’s disease. Mov Disord 27:84–89 21994070 10.1002/mds.23895

[5] Bhatt H, Pieruccini-Faria F, Almeida QJ (2013) Dynamics of turning sharpness influences freezing of gait in Parkinson’s disease. Parkinsonism Relat Disord 19:181–185 23083513 10.1016/j.parkreldis.2012.09.006

[6] Bohnen NI, Albin RL (2011) The cholinergic system and Parkinson disease. Behav Brain Res 221:564–573 20060022 10.1016/j.bbr.2009.12.048 PMC2888997

[7] Bohnen NI, Yarnall AJ, Weil RS, Moro E, Moehle MS, Borghammer P, Bedard MA, Albin RL (2022) Cholinergic system changes in Parkinson's disease: emerging therapeutic approaches. Lancet Neurol 10.1016/S1474-4422(21)00377-X PMC8985079 35131038

[8] Bouchouras G, Sofianidis G, Kotis K (2025) Predicting freezing of gait in Parkinson's Disease: A Machine-Learning-Based Approach in ON and OFF Medication States. J Clin Med 14 10.3390/jcm14062120 PMC11942655 40142927

[9] Bourilhon J, Olivier C, You H, Collomb-Clerc A, Grabli D, Belaid H, Mullie Y, François C, Czernecki V, Lau B, Pérez-García F, Bardinet E, Fernandez-Vidal S, Karachi C, Welter ML (2022) Pedunculopontine and cuneiform nuclei deep brain stimulation for severe gait and balance disorders in Parkinson’s disease: interim results from a randomized double-blind clinical trial. J Parkinsons Dis 12:639–653 34744048 10.3233/JPD-212793

[10] Cao Z, John AR, Chen HT, Martens KE, Georgiades M, Gilat M, Nguyen HT, Lewis SJG, Lin CT (2021) Identification of EEG dynamics during freezing of gait and voluntary stopping in patients with Parkinson’s disease. IEEE Trans Neural Syst Rehabil Eng 29:1774–1783 34428144 10.1109/TNSRE.2021.3107106

[11] Chan HL, Chen RS, Kuo CC, Chen YT, Liaw JW, Liao GS, Lin WT, Chien SH, Chang YJ (2024) Laser-light cueing shoes with integrated foot pressure and inertial sensing for investigating the impact of visual cueing on gait characteristics in Parkinson’s disease individuals. Front Bioeng Biotechnol 12:1334403 38357707 10.3389/fbioe.2024.1334403 PMC10865238

[12] Chou KL, Kanel P, van Emde Boas M, Roytman S, Carli G, Albin RL, Bohnen NI (2025) Cholinergic system changes in dopa-unresponsive freezing of gait in Parkinson’s disease. Mov Disord 40:1584–1594 40219650 10.1002/mds.30196 PMC12353882

[13] Chung KA, Carlson NE, Nutt JG (2005) Short-term paroxetine treatment does not alter the motor response to levodopa in PD. Neurology 64:1797–1798 15911816 10.1212/01.WNL.0000161841.41885.80

[14] Cockx HM, Oostenveld R, Flórez RY, Bloem BR, Cameron IGM, van Wezel RJA (2024) Freezing of gait in Parkinson's disease is related to imbalanced stopping-related cortical activity. Brain Commun 6:fcae259 10.1093/braincomms/fcae259 PMC11369826 39229492

[15] Denk D, Herman T, Zoetewei D, Ginis P, Brozgol M, Cornejo Thumm P, Decaluwe E, Ganz N, Palmerini L, Giladi N, Nieuwboer A, Hausdorff JM (2022) Daily-living freezing of gait as quantified using wearables in people With Parkinson disease: comparison with self-report and provocation tests. Phys Ther 102 10.1093/ptj/pzac129 PMC10071496 36179090

[16] Deuschl G, Herzog J, Kleiner-Fisman G, Kubu C, Lozano AM, Lyons KE, Rodriguez-Oroz MC, Tamma F, Tröster AI, Vitek JL, Volkmann J, Voon V (2006) Deep brain stimulation: postoperative issues. Mov Disord 21(Suppl 14):S219-237 16810719 10.1002/mds.20957

[17] Doyle LM, Kühn AA, Hariz M, Kupsch A, Schneider GH, Brown P (2005) Levodopa-induced modulation of subthalamic beta oscillations during self-paced movements in patients with Parkinson’s disease. Eur J Neurosci 21:1403–1412 15813950 10.1111/j.1460-9568.2005.03969.x

[18] Economou K, Quek D, MacDougall H, Lewis SJG, Ehgoetz Martens KA (2021) Heart rate changes prior to freezing of gait episodes are related to anxiety. J Parkinsons Dis 11:271–282 33074191 10.3233/JPD-202146

[19] Factor SA, Weinshenker D, McKay JL (2025) A possible pathway to freezing of gait in Parkinson’s disease. J Parkinsons Dis 15:282–290 39973500 10.1177/1877718X241308487 PMC13347428

[20] Faerman MV, Cole C, Van Ooteghem K, Cornish BF, Howe EE, Siu V, Norouzian P, Black A, Fraser JE, Grimes DA, Jog M, Kwan D, Lang AE, Lawrence-Dewar JM, Levine B, Marras C, Masellis M, McIlroy WE, McLaughlin PM, Montero-Odasso M, Orange JB, Peltsch AJ, Pieruccini-Faria F, Roberts AC, Sarquis-Adamson Y, Steeves TDL, Tan B, Troyer AK, Martens KAE (2025) Motor, affective, cognitive, and perceptual symptom changes over time in individuals with Parkinson’s disease who develop freezing of gait. J Neurol 272:321 40198411 10.1007/s00415-025-13034-y

[21] Ferraye MU, Debû B, Fraix V, Goetz L, Ardouin C, Yelnik J, Henry-Lagrange C, Seigneuret E, Piallat B, Krack P, Le Bas JF, Benabid AL, Chabardès S, Pollak P (2010) Effects of pedunculopontine nucleus area stimulation on gait disorders in Parkinson’s disease. Brain 133:205–214 19773356 10.1093/brain/awp229

[22] Giladi N, McMahon D, Przedborski S, Flaster E, Guillory S, Kostic V, Fahn S (1992) Motor blocks in Parkinson’s disease. Neurology 42:333–339 1736161 10.1212/wnl.42.2.333

[23] Giladi N, Shabtai H, Simon ES, Biran S, Tal J, Korczyn AD (2000) Construction of freezing of gait questionnaire for patients with Parkinsonism. Parkinsonism Relat Disord 6:165–170 10817956 10.1016/s1353-8020(99)00062-0

[24] Gilat M (2019) How to annotate freezing of gait from video: a standardized method using open-source software. J Parkinsons Dis 9:821–824 31524181 10.3233/JPD-191700

[25] Gilat M, Nonnekes J, Factor SA, Bloem BR, Nutt JG, Giladi N, Hallett M, Nieuwboer A, Horak FB, Weiss D, Cubo E, Zoetewei D, Moreau C, Jeon B, Virmani T, Hausdorff JM, Fasano A, Lewis SJG (2026) An updated definition of freezing of gait. Nat Rev Neurol 10.1038/s41582-025-01179-3 41513745

[26] Gilat M, Shine JM, Walton CC, O’Callaghan C, Hall JM, Lewis SJG (2015) Brain activation underlying turning in Parkinson’s disease patients with and without freezing of gait: a virtual reality fMRI study. NPJ Parkinsons Dis 1:15020 28725687 10.1038/npjparkd.2015.20 PMC5516618

[27] Ginis P, Heremans E, Ferrari A, Bekkers EMJ, Canning CG, Nieuwboer A (2017) External input for gait in people with Parkinson’s disease with and without freezing of gait: one size does not fit all. J Neurol 264:1488–1496 28653213 10.1007/s00415-017-8552-6

[28] Ginis P, Nackaerts E, Nieuwboer A, Heremans E (2018) Cueing for people with Parkinson’s disease with freezing of gait: a narrative review of the state-of-the-art and novel perspectives. Ann Phys Rehabil Med 61:407–413 28890341 10.1016/j.rehab.2017.08.002

[29] Goetz CG, Tilley BC, Shaftman SR, Stebbins GT, Fahn S, Martinez-Martin P, Poewe W, Sampaio C, Stern MB, Dodel R, Dubois B, Holloway R, Jankovic J, Kulisevsky J, Lang AE, Lees A, Leurgans S, LeWitt PA, Nyenhuis D, Olanow CW, Rascol O, Schrag A, Teresi JA, van Hilten JJ, LaPelle N (2008) Movement Disorder Society-sponsored revision of the Unified Parkinson’s Disease Rating Scale (MDS-UPDRS): scale presentation and clinimetric testing results. Mov Disord 23:2129–2170 19025984 10.1002/mds.22340

[30] Goh L, Paul SS, Canning CG, Ehgoetz Martens KA, Song J, Campoy SL, Allen NE (2022) The Ziegler test is reliable and valid for measuring freezing of gait in people with Parkinson disease. Phys Ther 102 10.1093/ptj/pzac122 PMC10071484 36130220

[31] Hall JM, Shine JM, O’Callaghan C, Walton CC, Gilat M, Naismith SL, Lewis SJ (2015) Freezing of gait and its associations in the early and advanced clinical motor stages of Parkinson’s Disease: a cross-sectional study. J Parkinsons Dis 5:881–891 26444088 10.3233/JPD-150581

[32] Harrison EC, Horin AP, Earhart GM (2018) Internal cueing improves gait more than external cueing in healthy adults and people with Parkinson disease. Sci Rep 8:15525 30341367 10.1038/s41598-018-33942-6 PMC6195608

[33] Henderson EJ, Lord SR, Brodie MA, Gaunt DM, Lawrence AD, Close JC, Whone AL, Ben-Shlomo Y (2016) Rivastigmine for gait stability in patients with Parkinson’s disease (ReSPonD): a randomised, double-blind, placebo-controlled, phase 2 trial. Lancet Neurol 15:249–258 26795874 10.1016/S1474-4422(15)00389-0

[34] Henderson J, Yiannikas C, Graham JS (1992) Effect of ritanserin, a highly selective 5-HT2 receptor antagonist, on Parkinson’s disease. Clin Exp Neurol 29:277–282 1343870

[35] Herman T, Barer Y, Bitan M, Sobol S, Giladi N, Hausdorff JM (2023) A meta-analysis identifies factors predicting the future development of freezing of gait in Parkinson’s disease. NPJ Parkinsons Dis 9:158 38049430 10.1038/s41531-023-00600-2 PMC10696025

[36] Hillel I, Gazit E, Nieuwboer A, Avanzino L, Rochester L, Cereatti A, Croce Ud D, Rikkert MO, Bloem BR, Pelosin E, Del Din S, Ginis P, Giladi N, Mirelman A, Hausdorff JM (2019) Is every-day walking in older adults more analogous to dual-task walking or to usual walking? Elucidating the gaps between gait performance in the lab and during 24/7 monitoring. Eur Rev Aging Phys Act 16:6 31073340 10.1186/s11556-019-0214-5 PMC6498572

[37] Hulzinga F, Nieuwboer A, Dijkstra BW, Mancini M, Strouwen C, Bloem BR, Ginis P (2020) The new freezing of gait questionnaire: unsuitable as an outcome in clinical trials? Mov Disord Clin Pract 7:199–205 32071940 10.1002/mdc3.12893 PMC7011794

[38] Iacono RP, Kuniyoshi SM, Ahlman JR, Zimmerman GJ, Maeda G, Pearlstein RD (1997) Concentrations of indoleamine metabolic intermediates in the ventricular cerebrospinal fluid of advanced Parkinson’s patients with severe postural instability and gait disorders. J Neural Transm (Vienna) 104:451–459 9295177 10.1007/BF01277663

[39] Jankovic J (2009) Atomoxetine for freezing of gait in Parkinson disease. J Neurol Sci 284:177–178 19361809 10.1016/j.jns.2009.03.022

[40] Klocke P, Loeffler MA, Lewis SJG, Gharabaghi A, Weiss D (2025) Could adaptive deep brain stimulation treat freezing of gait in Parkinson’s disease? J Neurol 272:267 40072634 10.1007/s00415-025-13000-8 PMC11903562

[41] Kratochvil CJ, Vaughan BS, Harrington MJ, Burke WJ (2003) Atomoxetine: a selective noradrenaline reuptake inhibitor for the treatment of attention-deficit/hyperactivity disorder. Expert Opin Pharmacother 4:1165–1174 12831341 10.1517/14656566.4.7.1165

[42] Kühn AA, Kempf F, Brücke C, Gaynor Doyle L, Martinez-Torres I, Pogosyan A, Trottenberg T, Kupsch A, Schneider GH, Hariz MI, Vandenberghe W, Nuttin B, Brown P (2008) High-frequency stimulation of the subthalamic nucleus suppresses oscillatory beta activity in patients with Parkinson’s disease in parallel with improvement in motor performance. J Neurosci 28:6165–6173 18550758 10.1523/JNEUROSCI.0282-08.2008 PMC6670522

[43] Kühn AA, Tsui A, Aziz T, Ray N, Brücke C, Kupsch A, Schneider GH, Brown P (2009) Pathological synchronisation in the subthalamic nucleus of patients with Parkinson’s disease relates to both bradykinesia and rigidity. Exp Neurol 215:380–387 19070616 10.1016/j.expneurol.2008.11.008

[44] Lewis SJ, Barker RA (2009) A pathophysiological model of freezing of gait in Parkinson’s disease. Parkinsonism Relat Disord. 15(5):333–8. 10.1016/j.parkreldis.2008.08.006 18930430 10.1016/j.parkreldis.2008.08.006

[45] Lewis SJG, Factor SA, Giladi N, Hallett M, Nieuwboer A, Nutt JG, Przedborski S, Papa SM (2022) Addressing the challenges of clinical research for freezing of gait in Parkinson’s disease. Mov Disord 37:264–267 34939228 10.1002/mds.28837 PMC8840955

[46] Liu Y, Zhang G, Tarolli CG, Hristov R, Jensen-Roberts S, Waddell EM, Myers TL, Pawlik ME, Soto JM, Wilson RM, Yang Y, Nordahl T, Lizarraga KJ, Adams JL, Schneider RB, Kieburtz K, Ellis T, Dorsey ER, Katabi D (2022) Monitoring gait at home with radio waves in Parkinson's disease: a marker of severity, progression, and medication response. Sci Transl Med 14:eadc9669 10.1126/scitranslmed.adc9669 PMC12853226 36130014

[47] Lizárraga KJ, Gnanamanogaran B, Al-Ozzi TM, Cohn M, Tomlinson G, Boutet A, Elias GJB, Germann J, Soh D, Kalia SK, Hodaie M, Munhoz RP, Marras C, Hutchison WD, Lozano AM, Lang AE, Fasano A (2022) Lateralized Subthalamic Stimulation for Axial Dysfunction in Parkinson's Disease: A Randomized Trial. Mov Disord 10.1002/mds.28953 35156734

[48] Lohnes CA, Earhart GM (2011) The impact of attentional, auditory, and combined cues on walking during single and cognitive dual tasks in Parkinson disease. Gait Posture 33:478–483 21273075 10.1016/j.gaitpost.2010.12.029

[49] Lopez-Delgado IE, Navarro-Lopez V, Grandas-Perez F, Godino-Llorente JI, Grajal J (2026) Radar network for gait monitoring: technology and validation. IEEE Trans Biomed Eng 73:393–403 40577290 10.1109/TBME.2025.3583785

[50] McKay LJ, Goldstein FC, Sommerfeld B, Bernhard D, Parra PS, Factor SA (2019) Freezing of gait can persist after an acute levodopa challenge in Parkinson’s disease. NPJ Parkinsons Dis 5:25 31799377 10.1038/s41531-019-0099-z PMC6874572

[51] Lv L, Zhang H, Tan X, Long Z, Qin L, Bai R, Xiao Q, Wu Z, Hu S, Tan C, Liao H, Yan W, Tang B, Ren F, Wang C (2022) Associated factors and abnormal dorsal raphe nucleus connectivity patterns of freezing of gait in Parkinson’s disease. J Neurol 269:6452–6466 35933494 10.1007/s00415-022-11294-6

[52] Maidan I, Plotnik M, Mirelman A, Weiss A, Giladi N, Hausdorff JM (2010) Heart rate changes during freezing of gait in patients with Parkinson’s disease. Mov Disord 25:2346–2354 20721914 10.1002/mds.23280 PMC2964413

[53] Mancini M, Bloem BR, Horak FB, Lewis SJG, Nieuwboer A, Nonnekes J (2019) Clinical and methodological challenges for assessing freezing of gait: Future perspectives. Mov Disord. 34(6):783–790. 10.1002/mds.27709 31046191 10.1002/mds.27709 PMC7105152

[54] Mancini M, Hausdorff JM, Pelosin E, Bonato P, Camicioli R, Ellis TD, Klucken J, Gifford L, Fasano A, Nieuwboer A, Kopil C, Klapper K, Kirsch L, Dexter DT, Fuest R, Miller V, Asis A, Müller ML, Stephenson D, Mirelman A (2025) A framework to standardize gait study protocols in Parkinson’s disease. J Parkinsons Dis 15:129–139 40007168 10.1177/1877718X241305626 PMC13347425

[55] Mancini M, McKay JL, Cockx H, D’Cruz N, Esper CD, Filtjens B, Heimler B, MacKinnon CD, Palmerini L, Roerdink M, Young WR, Hausdorff JM (2025) Technology for measuring freezing of gait: current state of the art and recommendations. J Parkinsons Dis 15:19–40 39973491 10.1177/1877718X241301065 PMC13347410

[56] Mancini M, Priest KC, Nutt JG, Horak FB (2012) Quantifying freezing of gait in Parkinson’s disease during the instrumented timed up and go test. Annu Int Conf IEEE Eng Med Biol Soc 2012:1198–1201 23366112 10.1109/EMBC.2012.6346151 PMC4140195

[57] Matar E, Shine JM, Gilat M, Ehgoetz Martens KA, Ward PB, Frank MJ, Moustafa AA, Naismith SL, Lewis SJG (2019) Identifying the neural correlates of doorway freezing in Parkinson’s disease. Hum Brain Mapp 40:2055–2064 30637883 10.1002/hbm.24506 PMC6865388

[58] McKay JL, Nye J, Goldstein FC, Sommerfeld B, Smith Y, Weinshenker D, Factor SA (2023) Levodopa responsive freezing of gait is associated with reduced norepinephrine transporter binding in Parkinson?s disease. Neurobiol Dis 179 10.1016/j.nbd.2023.106048 PMC13001024 36813207

[59] Mirelman A, Bonato P, Camicioli R, Ellis TD, Giladi N, Hamilton JL, Hass CJ, Hausdorff JM, Pelosin E, Almeida QJ (2019) Gait impairments in Parkinson’s disease. Lancet Neurol 18:697–708 30975519 10.1016/S1474-4422(19)30044-4

[60] Moreau C, Defebvre L, Destée A, Bleuse S, Clement F, Blatt JL, Krystkowiak P, Devos D (2008) STN-DBS frequency effects on freezing of gait in advanced Parkinson disease. Neurology 71:80–84 18420482 10.1212/01.wnl.0000303972.16279.46

[61] Moro E, Hamani C, Poon YY, Al-Khairallah T, Dostrovsky JO, Hutchison WD, Lozano AM (2010) Unilateral pedunculopontine stimulation improves falls in Parkinson’s disease. Brain 133:215–224 19846583 10.1093/brain/awp261

[62] Morris TR, Cho C, Dilda V, Shine JM, Naismith SL, Lewis SJ, Moore ST (2012) A comparison of clinical and objective measures of freezing of gait in Parkinson’s disease. Parkinsonism Relat Disord 18:572–577 22445248 10.1016/j.parkreldis.2012.03.001

[63] Muller M, Contarino MF (2025) Double fault: artifacts produced by physical activity can impact the efficacy of chronic local field potential recordings and adaptive stimulation. Mov Disord Clin Pract 10.1002/mdc3.70480 PMC13172738 41424237

[64] Nieuwboer A, Giladi N (2013) Characterizing freezing of gait in Parkinson’s disease: models of an episodic phenomenon. Mov Disord 28:1509–1519 24132839 10.1002/mds.25683

[65] Nieuwboer A, Rochester L, Herman T, Vandenberghe W, Emil GE, Thomaes T, Giladi N (2009) Reliability of the new freezing of gait questionnaire: agreement between patients with Parkinson’s disease and their carers. Gait Posture 30:459–463 19660949 10.1016/j.gaitpost.2009.07.108

[66] Nonnekes J, Janssen AM, Mensink SH, Oude Nijhuis LB, Bloem BR, Snijders AH (2014) Short rapid steps to provoke freezing of gait in Parkinson’s disease. J Neurol 261:1763–1767 24957299 10.1007/s00415-014-7422-8

[67] Nutt JG, Bloem BR, Giladi N, Hallett M, Horak FB, Nieuwboer A (2011) Freezing of gait: moving forward on a mysterious clinical phenomenon. Lancet Neurol 10:734–744 21777828 10.1016/S1474-4422(11)70143-0 PMC7293393

[68] Pallavi P, Raghuvanshi A, Kumar SD, Patel N, Kanetkar M, Chhatlani R, Rana M, Betai S, Rajan R, Lahiri U (2025) On-demand cueing sensitive to step variability: understanding its impact on gait of individuals with Parkinson’s disease. IEEE J Transl Eng Health Med 13:183–192 40657531 10.1109/JTEHM.2025.3563381 PMC12251033

[69] Pardoel S, Shalin G, Nantel J, Lemaire ED, Kofman J (2021) Early detection of freezing of gait during walking using inertial measurement unit and plantar pressure distribution data. Sensors (Basel) 21 10.3390/s21062246 PMC8004667 33806984

[70] Picillo M, Lozano AM, Kou N, Puppi Munhoz R, Fasano A (2016) Programming deep brain stimulation for Parkinson’s disease: The Toronto Western Hospital Algorithms. Brain Stimul 9:425–437 26968806 10.1016/j.brs.2016.02.004

[71] Revuelta GJ, Embry A, Elm JJ, Gregory C, Delambo A, Kautz S, Hinson VK (2015) Pilot study of atomoxetine in patients with Parkinson’s disease and dopa-unresponsive freezing of gait. Transl Neurodegener 4:24 26693006 10.1186/s40035-015-0047-8 PMC4676139

[72] Rocha PA, Porfírio GM, Ferraz HB, Trevisani VF (2014) Effects of external cues on gait parameters of Parkinson’s disease patients: a systematic review. Clin Neurol Neurosurg 124:127–134 25043443 10.1016/j.clineuro.2014.06.026

[73] Scully AE, Tan D, Oliveira BIR, Hill KD, Clark R, Pua YH (2023) Scoring festination and gait freezing in people with Parkinson’s: the freezing of gait severity tool-revised. Physiother Res Int 28:e2016 37199289 10.1002/pri.2016

[74] Shine JM, Moore ST, Bolitho SJ, Morris TR, Dilda V, Naismith SL, Lewis SJ (2012) Assessing the utility of freezing of gait questionnaires in Parkinson’s disease. Parkinsonism Relat Disord 18:25–29 21872523 10.1016/j.parkreldis.2011.08.002

[75] Shine JM, van den Brink RL, Hernaus D, Nieuwenhuis S, Poldrack RA (2018) Catecholaminergic manipulation alters dynamic network topology across cognitive states. Netw Neurosci 2:381–396 30294705 10.1162/netn_a_00042 PMC6145851

[76] Sousa M, Tinkhauser G (2026) Being guided by your brain or by your heart? Challenges in adaptive Deep Brain Stimulation. Mov Disord Clin Pract 13:287–290 40878349 10.1002/mdc3.70337 PMC12999026

[77] Starr PA, Shivacharan RS, Goldberg E, Tröster AI, House PA, Giroux ML, Hebb AO, Whiting DM, Leichliter TA, Ostrem JL, Metman LV, Sani S, Karl JA, Siddiqui MS, Tatter SB, Haq IU, Machado AG, Gostkowski M, Tagliati M, Mamelak AN, Okun MS, Foote KD, Moguel-Cobos G, Ponce FA, Pahwa R, Lyons K, Buetefisch CM, Gross RE, Luca CC, Jagid JR, Revuelta GJ, Takacs I, Pourfar MH, Mogilner AY, Duker AP, Mandybur GT, Rosenow JM, Zadikoff C, Khandhar SM, Sedrak M, Phibbs FT, Neimat J, Durphy J, Ramirez-Zamora A, Pilitsis JG, Uitti RJ, Wharen R, Park MC, Vitek JL (2025) Five-year outcomes from deep brain stimulation of the subthalamic nucleus for Parkinson disease. JAMA Neurol 82:1181–1190 40952750 10.1001/jamaneurol.2025.3373 PMC12439180

[78] Suteerawattananon M, Morris GS, Etnyre BR, Jankovic J, Protas EJ (2004) Effects of visual and auditory cues on gait in individuals with Parkinson’s disease. J Neurol Sci 219:63–69 15050439 10.1016/j.jns.2003.12.007

[79] Takahashi M, Tabu H, Ozaki A, Hamano T, Takeshima T (2019) Antidepressants for depression, apathy, and gait instability in Parkinson’s Disease: a multicenter randomized study. Intern Med 58:361–368 30146591 10.2169/internalmedicine.1359-18 PMC6395136

[80] Tanaka Y, Tsuboi T, Watanabe H, Nakatsubo D, Maesawa S, Kato S, Kajita Y, Sato M, Oodake R, Hattori M, Yamamoto M, Wakabayashi T, Katsuno M, Sobue G (2020) Longitudinal speech change after subthalamic nucleus deep brain stimulation in Parkinson’s disease patients: a 2-year prospective study. J Parkinsons Dis 10:131–140 31884493 10.3233/JPD-191798

[81] Taylor NL, Wainstein G, Quek D, Lewis SJG, Shine JM, Ehgoetz Martens KA (2022) The contribution of noradrenergic activity to anxiety-induced freezing of gait. Mov Disord 37:1432–1443 35384055 10.1002/mds.28999 PMC9540856

[82] Tosserams A, Bloem BR, Ehgoetz Martens KA, Helmich RC, Kessels RPC, Shine JM, Taylor NL, Wainstein G, Lewis SJG, Nonnekes J (2023) Modulating arousal to overcome gait impairments in Parkinson’s disease: how the noradrenergic system may act as a double-edged sword. Transl Neurodegener 12:15 36967402 10.1186/s40035-023-00347-z PMC10040128

[83] Tosserams A, Fasano A, Gilat M, Factor SA, Giladi N, Lewis SJG, Moreau C, Bloem BR, Nieuwboer A, Nonnekes J (2025) Management of freezing of gait - mechanism-based practical recommendations. Nat Rev Neurol 10.1038/s41582-025-01079-6 40169855

[84] van der Laan M, Rietberg MB, van der Ent M, Waardenburg F, de Groot V, Nonnekes J, van Wegen EEH (2025) User experiences of the Cue2walk smart cueing device for freezing of gait in people with Parkinson's disease. Sensors (Basel) 25 10.3390/s25154702 PMC12349309 40807870

[85] van Nuenen BF, Esselink RA, Munneke M, Speelman JD, van Laar T, Bloem BR (2008) Postoperative gait deterioration after bilateral subthalamic nucleus stimulation in Parkinson’s disease. Mov Disord 23:2404–2406 18951532 10.1002/mds.21986

[86] Vitorio R, Morris R, Graham L, Das J, Walker R, McDonald C, Mancini M, Stuart S (2025) Effects of internal and external cues on brain activity and gait in Parkinson’s Disease: findings from BARC-PD. Neurorehabil Neural Repair 39:826–838 40652355 10.1177/15459683251351876 PMC12476477

[87] Wang F, Skubic M, Rantz M, Cuddihy PE (2014) Quantitative gait measurement with pulse-Doppler radar for passive in-home gait assessment. IEEE Trans Biomed Eng 61:2434–2443 24771566 10.1109/TBME.2014.2319333 PMC4238914

[88] Wang SP, Wu T, Cai YJ, Yu YQ, Chen XW, Wang LS (2023) Neuromelanin magnetic resonance imaging of substantia nigra and locus coeruleus in Parkinson's disease with freezing of gait. Front Aging Neurosci 15 10.3389/fnagi.2023.1060935 PMC9932285 36819729

[89] Weintraub D, Mavandadi S, Mamikonyan E, Siderowf AD, Duda JE, Hurtig HI, Colcher A, Horn SS, Nazem S, Ten Have TR, Stern MB (2010) Atomoxetine for depression and other neuropsychiatric symptoms in Parkinson disease. Neurology 75:448–455 20679638 10.1212/WNL.0b013e3181ebdd79 PMC2918470

[90] Weiss D, Walach M, Meisner C, Fritz M, Scholten M, Breit S, Plewnia C, Bender B, Gharabaghi A, Wachter T, Kruger R (2013) Nigral stimulation for resistant axial motor impairment in Parkinson’s disease? A randomized controlled trial. Brain 136:2098–2108 23757762 10.1093/brain/awt122 PMC3692032

[91] Wilkins KB, Petrucci MN, Lambert EF, Melbourne JA, Gala AS, Akella P, Parisi L, Cui C, Kehnemouyi YM, Hoffman SL, Aditham S, Diep C, Dorris HJ, Parker JE, Herron JA, Bronte-Stewart HM (2025) Beta burst-driven adaptive deep brain stimulation for gait impairment and freezing of gait in Parkinson's disease. Brain Commun 7:fcaf266 10.1093/braincomms/fcaf266 PMC12268161 40677701

[92] Witt K, Daniels C, Volkmann J (2012) Factors associated with neuropsychiatric side effects after STN-DBS in Parkinson’s disease. Parkinsonism Relat Disord 18(Suppl 1):S168-170 22166423 10.1016/S1353-8020(11)70052-9

[93] Zhang W, Han Y, Shi Y, Yan S, Song W, Cui G, Xiang J (2023) Effects of wearable visual cueing on gait pattern and stability in patients with Parkinson’s disease. Front Neurol 14:1077871 37064198 10.3389/fneur.2023.1077871 PMC10091618

[94] Ziegler K, Schroeteler F, Ceballos-Baumann AO, Fietzek UM (2010) A new rating instrument to assess festination and freezing gait in Parkinsonian patients. Mov Disord 25:1012–1018 20310009 10.1002/mds.22993

